# Characteristics of Moyamoya Syndrome in Sickle-Cell Disease by Magnetic Resonance Angiography: An Adult-Cohort Study

**DOI:** 10.3389/fneur.2019.00015

**Published:** 2019-01-22

**Authors:** Paul Kauv, Noémie Gaudré, Jérôme Hodel, Titien Tuilier, Anoosha Habibi, Catherine Oppenheim, Myriam Edjlali, Dominique Hervé, David Calvet, Pablo Bartolucci

**Affiliations:** ^1^Department of Neuroradiology, APHP, Hôpital Henri Mondor, UPEC, Créteil, France; ^2^Unité des Maladies Génétiques du Globule Rouge, Sickle-Cell Referral Center, Internal Medicine, APHP, Hôpital Henri Mondor, UPEC, Créteil, France; ^3^Department of Neuroradiology, Hôpital Sainte-Anne, Université Paris Descartes, INSERM U894, Paris, France; ^4^Department of Neurology, APHP, Hôpital Lariboisière, Paris, France; ^5^Department of Neurology, Hôpital Sainte-Anne, Paris, France; ^6^INSERM-U955, IMRB, Team 2: Transfusion et Maladies du Globule Rouge, Laboratoire d'Excellence, GRex, Créteil, France

**Keywords:** magnetic resonance angiography, magnetic resonance imaging, moyamoya syndrome, sickle-cell disease, time-of-flight

## Abstract

**Background:** Sickle cell disease (SCD) can be complicated by moyamoya syndrome. Brain magnetic resonance angiography (MRA) is a non-invasive method to diagnose this syndrome and, steno-occlusion and moyamoya vessels (MMV) scores have been proposed to evaluate its severity. Previous studies of SCD moyamoya syndrome did not evaluate the severity according to MRA scores. The objective was to assess the characteristics of moyamoya syndrome in an adult cohort of SCD using these MRA scores.

**Methods:** Twenty-five SCD patients with moyamoya syndrome were included using MRA with 3D time of flight technique. We evaluate steno-occlusion score for each hemisphere (range 0–10) from: steno-occlusion severity of internal carotid (ICA) (0–3), anterior cerebral (ACA) (0–3), middle cerebral (MCA) (0–2), and posterior cerebral (PCA) (0–2) arteries. MMV score for each hemisphere (range 0–5) depended from 5 MMV areas: (1) anterior communicating artery (2) basal ganglia (3) ICA/MCA (4) posterior communicating artery/PCA (5) basilar artery.

**Results:** Eight patients (32%) showed unilateral moyamoya syndrome. ICA steno-occlusion was involved in 22 patients (88%), MCA in 23 patients (92%), ACA in 9 patients (36%), and PCA in 3 patients (12%). MMV involved ACoA area in 10 patients (40%), basal ganglia in 13 patients (52%), PCoA/PCA in 10 patients (40%), MCA/ICA in 7 patients (28%), and BA in 1 patient (4%). Steno-occlusion and MMV mean hemisphere scores were 3.4/10 (± 1.42) and 1.6/5 (± 0.71), respectively.

**Conclusion:** Frequent unilateral moyamoya syndrome, uncommon PCA involvement and, moderate steno-occlusion and MMV scores seem to be features of SCD moyamoya syndrome. In future studies, MRA scores could be collected to assess the follow-up in these patients.

## Introduction

Sickle cell disease (SCD) of homozygous hemoglobin S (HbSS) may be complicated by moyamoya syndrome (1), an intracranial angiopathy defined by steno-occlusion of terminal portion of internal carotid artery (ICA) and development of collateral vessels, called moyamoya vessels (MMV) (2). Ischemic stroke is the most frequent cerebrovascular consequence of this angiopathy but hemorrhagic event can also occur (3, 4). The imaging characteristics of moyamoya syndrome in SCD patients are not well-known as opposed to moyamoya disease (MMD). Conventional angiography can be performed (1) to diagnose and evaluate the moyamoya angiopathy but brain magnetic resonance angiography (MRA) appears as a non-invasive alternative method (5–8). In MMD studies, a steno-occlusion score and a MMV score have been proposed to quantify the severity of the angiopathy by MRA (9, 10). Previous studies of SCD moyamoya syndrome using MRA on pediatric or/and adult population (3, 11–13) did not evaluate the severity of the angiopathy using these imaging MRA scores.

Therefore, we aimed to assess the characteristics of moyamoya syndrome in adult SCD using these MRA scores.

## Methods

### Study Population

Since January 2011, SCD adults (≥18 years) with previous history of neurological event (e.g., stroke, transient ischemic attack, seizures) and those who had a definite (previous abnormal transcranial doppler or MRA) or probable (doubtful stenosis or suboptimal previous vessel workup) intracranial angiopathy were enrolled in the prospective, multicenter, observational, cohort named PCDREP (Perfusion Cérébrale DREPanocytose), and had a MRA evaluation. This cohort received Ethics Committee (Comité de Protection des Personnes Île-de-France Saint-Louis) approval. Patients were enrolled after giving their written informed consent.

In this cohort, patients with moyamoya syndrome were selected for our study by an expert neuroradiologist (M.E.) according to imaging MRA criteria (14) and was defined as a steno-occlusion of portion of terminal ICA and/or proximal portions of anterior cerebral artery (ACA) or middle cerebral artery (MCA) and associated with abnormal vascular network (i.e., MMV) based on imaging time-of-flight MRA.

### MRA Protocol

All patients had a brain MRA on a 3.0-T MRI unit (Discovery MR750, GE HealthCare) using a multichannel head and neck coil. The protocol included a three-dimensional gradient recalled TOF sequence [parameters: repetition time (TR)/ echo time (TE) = 22/2.7 ms, flip angle = 15°, voxel = 0.6 × 0.8 × 1.2 mm, matrix = 352 × 256, field of view = 215 × 218 mm, scan thickness = 1.2 mm, number of slices = 144].

TOF images were reviewed by two neuroradiologists (P.K. and T.T.) unaware of clinical status. They assessed right and left cerebral hemisphere separately (i.e., they assessed 50 hemispheres from the 25 patients). Discordant readings were resolved by consensus.

### Steno-Occlusion Score

Using TOF images, steno-occlusion severity was assessed according to previous study (12) using a steno-occlusion score (range 0–10) for each hemisphere, defined as the sum of the severity of steno-occlusion of the ICA (0–3), ACA (0–3), MCA (0–2). and posterior cerebral (PCA) (0–2) arteries (Supplementary Table 1).

### MMV Score

Using TOF images, the presence of MMV was assessed according to a previous study (10) using a MMV score (range 0–5) for each hemisphere, depending of the involvement of the five collateral artery areas of the circle of Willis: (1) anterior communicating artery (ACoA) (2) basal ganglia (3) ICA/MCA (4) posterior communicating artery (PCoA)/PCA (5) basilar artery (BA) (10). For each area, collateral artery was scored as 1 if present or 0 if absent (Supplementary Figure 1).

### Statistical Analyses

The mean of steno-occlusion score and MMV score, and standard deviation (±) per hemisphere were calculated. Since moyamoya syndrome could be unilateral, if a hemisphere did not have steno-occlusion or MMV, i.e., considered as normal and therefore with a score of 0, it was excluded for the calculation of the mean score per hemisphere. Comparison of clinical data between patients with unilateral steno-occlusion and those with bilateral steno-occlusion was evaluated by Friedman tests. Correlation between steno-occlusion score and MMV score was evaluated with Pearson's test (Graphpad; Prism); *P* < 0.05 defined significance.

## Results

### Study Population

From January 2011 to October 2015, 74 SCD adult patients with intracranial angiopathy were enrolled in the PCDREP cohort. Among these patients, 25 patients (all SS genotype) had a moyamoya syndrome and were included in this study. Mean age was 32 (range 21–45) years and 65% were female. Patients were enrolled according to previous history of neurological event: 68% of patients had a history of stroke, either ischemic in 86%; 14% of the patients had hemorrhagic stroke; or 12% of the patients were enrolled because of abnormal transcranial doppler. Among the 25 patients, 40% had clinical history of seizure. All patients had a chronic blood-exchange–transfusion program to lower hemoglobin S (HbS) < 30%, except 2 patients since they had delayed hemolytic transfusion reactions and were changed to hydroxyurea alone; 18 patients had blood exchanged transfusion (BET) and hydroxyurea. None of the patients had bone allograft or surgical revascularization.

The 49 excluded patients (all SS genotype) with a mean age of 33 years had steno-occlusion without MMV and were symptomatic (28% had ischemic stroke, 14% had hemorrhagic stroke, and 15% had seizure).

### Steno-Occlusion of Intracranial Arteries and Steno-Occlusion Score

**Figure 1:**
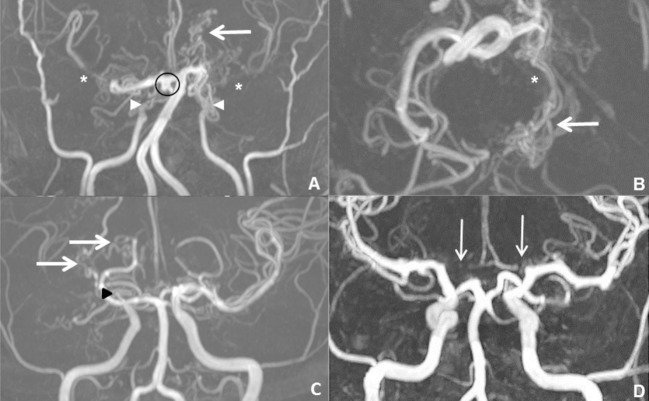
Examples of time-of-flight (TOF) evaluations in maximal intensity projection (MIP) of three patients with SCD moyamoya syndrome. **(A)** Coronal TOF images in a 27-year-old patient showed bilateral ICA steno-occlusions (arrowheads), with MCA discontinuity (^*^) and basal ganglia moyamoya vessels (MMV) (arrow). He also had a circle of Willis aneurysm (circle). **(B)** Axial TOF images demonstrated PCoA/PCA MMV (arrow) due to left PCA occlusion (^*^). For the left hemisphere and the right hemisphere, respectively, his MRA steno-occlusion score was 6/10 and 3/10, and MMV score was 3/5 and 2/5. **(C)** Another 28-year-old patient had unilateral steno-occlusion involving right MCA (arrowhead) which was invisible and he had right basal ganglia and MCA/ICA MMV (arrows). His right steno-occlusion and MMV scores were 3/10 and 2/10, respectively. **(D)** A 29-year-old patient revealed bilateral ACA stenosis (arrows) without ICA stenosis.

Among the 25 included patients, steno-occlusions were unilateral in 12 patients (44%) (Figure 1C) and bilateral in 13 patients (56%) (Figure 1A). ICA steno-occlusion was involved in 22 patients (88%), MCA in 23 patients (92%), ACA in 9 patients (36%), and PCA in 3 patients (12%) (Figure 1B, Table 1). These 3 patients with PCA steno-occlusion were combined with ICA, MCA, and ACA steno-occlusions. It is noteworthy that 2 patients had ACA steno-occlusion and 1 patient had MCA steno-occlusion, without ICA steno-occlusion (Figure 1D). Patients with unilateral steno-occlusion did not differ from those with bilateral steno-occlusion in terms of sex and age. The mean steno-occlusion score per hemisphere was 3.4/10 (±1.42) (range 1–6).

**Table 1:** Steno-occlusion score and MMV score in the 25 patients with unilateral or bilateral SCD moyamoya syndrome.

**Steno-occlusion**	**Number (%)**
**ARTERIES INVOLVED IN STENO-OCCLUSION (PER PATIENT**, ***N*** **=** **25)**	
ICA	22 (88%)
MCA	23 (92%)
ACA	9 (36%)
PCA	3 (12%)
**STENO-OCCLUSION SCORE (PER HEMISPHERE**, ***N*** **=** **50)**	
0	14 (28%)
1	5 (10%)
2	4 (8%)
3	12 (24%)
4	9 (18%)
5	3 (6%)
6	3 (6%)
**MMV**	**Number (%)**
**MMV AREAS INVOLVED (PER PATIENT**, ***N*** **=** **25)**	
ACoA	10 (40%)
Basal ganglia	13 (52%)
MCA/ICA	7 (28%)
PCoA/PCA	10 (40%)
BA	1 (4%)
**MMV SCORE (PER HEMISPHERE**, ***N*** **=** **50)**	
0	10 (20%)
1	22 (44%)
2	13 (26%)
3	5 (10%)

*ICA, internal carotid artery; MCA, middle cerebral artery; ACA, anterior cerebral artery; PCA, posterior cerebral artery; MMV, moyamoya vessels; ACoA, anterior communicating artery; PCoA, posterior communicating artery, BA, basilar artery*.

### MMV and MMV Score

Among the 25 patients, MMV involved ACoA area in 10 patients (40%), basal ganglia in 13 patients (52%), PCoA/PCA in 10 patients (40%), MCA/ICA in 7 patients (28%) and BA in 1 patient (4%) (Table 1). The mean MMV score per hemisphere was 1.6/5 (±0.71) (range 1–3).

### Steno-Occlusion of Intracranial Arteries and MMV

Among the 13 patients who had bilateral steno-occlusion, 10 had bilateral MMV and 3 had unilateral MMV. Among the 12 patients who had unilateral steno-occlusion, all had ipsilateral MMV: 4 had bilateral MMV and 8 had unilateral MMV (i.e., 32% off all the patients had unilateral moyamoya syndrome).

Steno-occlusion score correlated with MMV score (*r* = 0.5, *P* < 0.001).

Regarding previous neurological event, patients who had a history of stroke had a more severe angiopathy compared with those without (mean steno-occlusion score = 2.5 vs. 2.3 and mean MMV score = 1.4 vs. 0.9, respectively). In those who had a stroke, angiopathy was more severe in hemorrhagic stroke compared to ischemic stroke (mean steno-occlusion score = 3.3 vs. 2.5 and mean MMV = 1.6 vs. 1.4, respectively).

## Discussion

Previous studies of SCD moyamoya syndrome was focused on pediatric population (3, 13) and was poorly described in adulthood from case reports (11, 12). Moreover, the severity of the disease has never been evaluated with quantitative method as imaging MRA scores. These scores could be useful for an objective follow-up of SCD patients with moyamoya syndrome. Computed tomography or conventional angiography could be alternative evaluations; however, conversely to TOF MRA, the first requires intravenous contrast and the second requires arterial catheterism which is invasive.

In this study, we found that moyamoya syndrome was unilateral in 32% of adult SCD patients which is more frequent than what has been observed in MMD patients (10–20% of cases) (14). These results are in accordance with a previous MRA study in SCD moyamoya syndrome in which unilateral steno-occlusion was observed in 31% of cases (13). To explain this frequent unilateral moyamoya syndrome in our patients, the treatment of SCD to lower HbS could prevent the involvement of the contralateral normal side.

Regarding involved arteries, we found ICA and MCA were the most involved arteries as it was already observed in MMD patients (2). The involvement of PCA was uncommon (12%) and less frequent than what has been found in MMD (30%) (15). This uncommon PCA involvement in SCD moyamoya syndrome was also reported (6% of cases) in a previous study (13).

Regarding MRA scores, steno-occlusion score was less severe in our population of SCD moyamoya syndrome, as attested by our results with a mean of 3.4/10, than in MMD with a score from 4.6/10 up to 5/10 showed in two previous studies (9, 10). To explain this difference, in SCD moyamoya syndrome, frequent unilateral steno-occlusion and thus frequent contralateral normal side could prevent the worsening of steno-occlusion side by a persistent flow. Conversely, in MMD, bilateral steno-occlusion is common and could not insure sufficient flow to prevent from the increase of steno-occlusion from one side to the other. The mean of MMV score of 1.6 was less severe in our SCD patients than what has been observed in MMD (score of 3.9/5) (10). Finally, the presence of a correlation between the steno-occlusion score and the MMV score was herein confirmed as it has been already found previously in MMD and in SCD moyamoya syndrome (3, 10).

However, this study had limitation since we did not compare our cohort of SCD moyamoya syndrome to moyamoya disease from the same study within same imaging platform and evaluation of radiologist.

## Conclusions

This study suggests that SCD moyamoya syndrome is characterized by frequent unilateral moyamoya syndrome, uncommon PCA involvement and moderate steno-occlusion and MMV scores. Steno-occlusion and MMV MRA scores could be useful in patient's follow-up to quantify the severity of this angiopathy.

## Author Contributions

PK, NG, TT, AH, DC, and PB: conception and design, or acquisition of data, or analysis and interpretation of data. PK, NG, DC, PB, ME, and DH: drafting the article or revising it critically for important intellectual content. DC and PB: final approval of the version to be published. PK, NG, TT, AH, JH, CO, DC, PB, ME, and DH: agreement to be accountable for all aspects of the work in ensuring that questions related to the accuracy or integrity of any part of the work are appropriately investigated and resolved.

### Conflict of Interest Statement

The authors declare that the research was conducted in the absence of any commercial or financial relationships that could be construed as a potential conflict of interest.

## Abbreviations

ACA: anterior cerebral artery
ACoA: anterior communicating artery
BA: basilar artery
BET: blood exchange transfusion
ICA: internal carotid artery
MCA: middle cerebral artery
MMV: moyamoya vessel
MRA: magnetic resonance angiography
PCA: posterior cerebral artery
PCoA: posterior communicating artery
PCDREP: perfusion cerebrale drépanocytose
TOF: time-of-flight
SCD: sickle cell disease.
