# Supplementary material for: Characteristics of Moyamoya Syndrome in Sickle-Cell Disease by Magnetic Resonance Angiography: An Adult-Cohort Study
Source: Front Neurol. 2019 Jan 22;10:15. doi: 10.3389/fneur.2019.00015 (PMC6349744; doi:10.3389/fneur.2019.00015)
Supplement: Supplementary file 1 [file Table_1.docx]

**Supplementary Table 1.** Steno-occlusion score evaluated by TOF images, per hemisphere (12)

| Item | Steno-occlusion score |
| --- | --- |
| ICA |  |
| Normal | 0 |
| Stenosis of C1 | 1 |
| Discontinuity of C1 signal | 2 |
| Invisible | 3 |
| MCA |  |
| Normal | 0 |
| Stenosis of M1 | 1 |
| Discontinuity of M1 signal | 2 |
| Invisible | 3 |
| ACA |  |
| Normal A2 and its distal | 0 |
| A2 and its distal signal decrease or loss | 1 |
| Invisible | 2 |
| PCA |  |
| Normal P2 and its distal | 0 |
| P2 and its distal signal decrease or loss | 1 |
| Invisible | 2 |
| Total (steno-occlusion score) | 0–10 |

ICA: internal carotid artery; MCA: middle cerebral artery; ACA: anterior cerebral artery; PCA; posterior cerebral artery. C1 is the ICA portion, distal to the posterior communicating artery. M1 is the horizontal portion of the MCA. A2 is the vertical portion of the ACA from the anterior communicating artery. P2 is the PCA portion around the midbrain from the posterior communicating artery.
