# Supplementary material for: Characteristics of Moyamoya Syndrome in Sickle-Cell Disease by Magnetic Resonance Angiography: An Adult-Cohort Study
Source: Front Neurol. 2019 Jan 22;10:15. doi: 10.3389/fneur.2019.00015 (PMC6349744; doi:10.3389/fneur.2019.00015)
Supplement: Supplementary file 2 [file Presentation_1.pptx]

## Slide 1
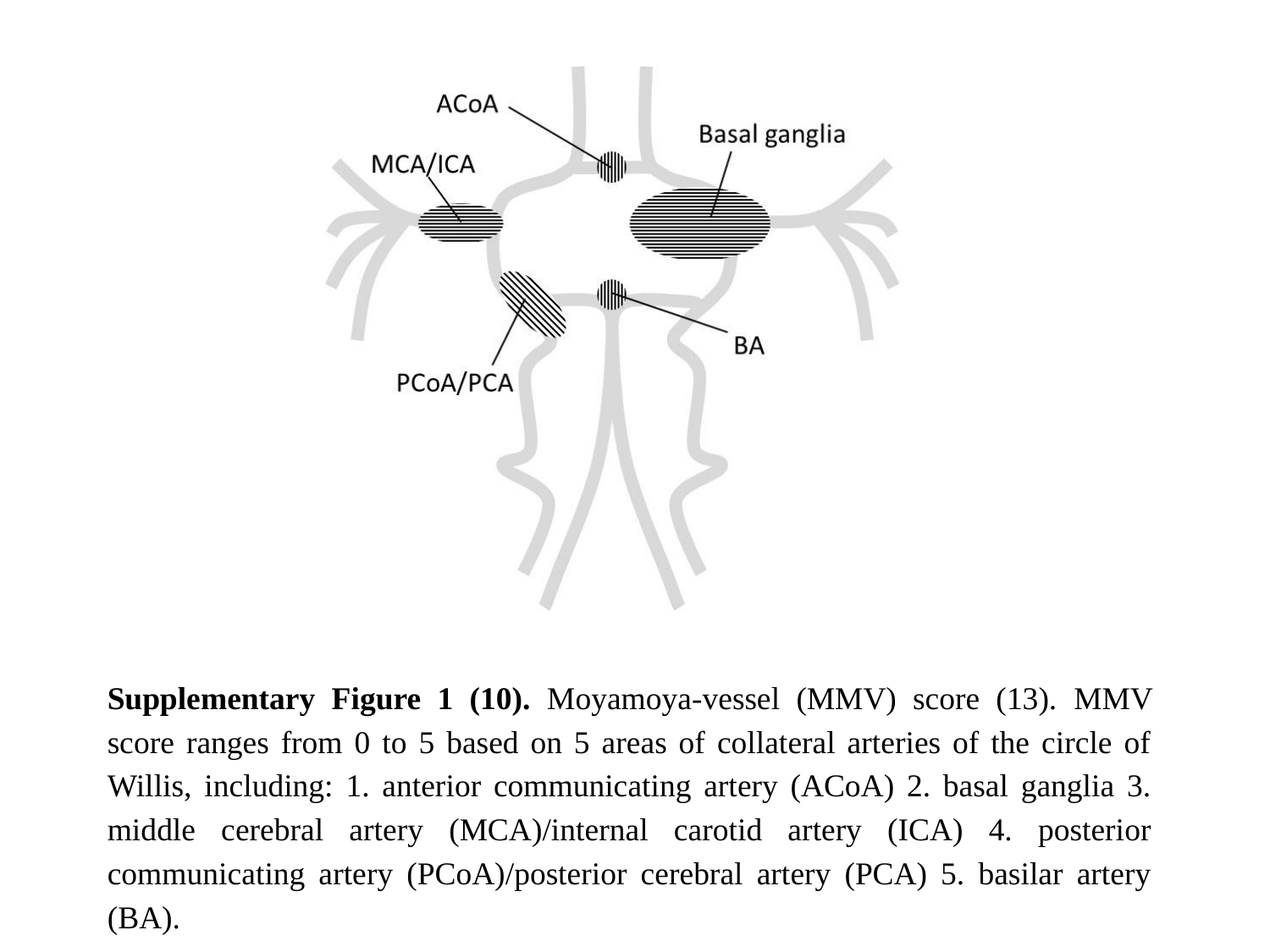

Supplementary Figure 1 (10). Moyamoya-vessel (MMV) score (13). MMV score ranges from 0 to 5 based on 5 areas of collateral arteries of the circle of Willis, including: 1. anterior communicating artery (ACoA) 2. basal ganglia 3. middle cerebral artery (MCA)/internal carotid artery (ICA) 4. posterior communicating artery (PCoA)/posterior cerebral artery (PCA) 5. basilar artery (BA).
